# Diagnostic Criteria and Genetic Basis of Polycystic Ovary Syndrome: A Narrative Review

**DOI:** 10.3390/metabo16040277

**Published:** 2026-04-19

**Authors:** María de los Angeles Cepero-González, Adriana Aguilar-Galarza, Víctor Manuel Rodríguez-García, Teresa García-Gasca, Ulisses Moreno Celis

**Affiliations:** 1 Facultad de Ciencias Naturales, Universidad Autónoma de Querétaro, Querétaro 76230, Mexico; ceperomaria1996@gmail.com (M.d.l.A.C.-G.); beatriz.aguilar@uaq.mx (A.A.-G.); tggasca@uaq.edu.mx (T.G.-G.); 2 Centro de Investigación e innovación en Nutrición y Salud, Universidad Autónoma de Querétaro, Querétaro 76230, Mexico; vmrodrigg@tec.mx; 3 Escuela de Ingeniería y Ciencias, Tecnologico de Monterrey, San Pablo, Querétaro 76130, Mexico

**Keywords:** polycystic ovary syndrome, polymorphisms, candidate genes, hyperandrogenism, ovarian dysfunction

## Abstract

This study reviews the main candidate genes involved in the pathophysiology of Polycystic Ovary Syndrome (PCOS). PCOS is a common endocrine–metabolic disorder in women of reproductive age, characterized by menstrual irregularity, hyperandrogenism, and polycystic ovarian morphology. It is associated with increased metabolic and cardiovascular risk and is a leading cause of infertility. Although its pathophysiology is not fully understood, alterations in the hypothalamic–pituitary–ovarian axis, insulin metabolism, and steroidogenesis have been described. Polymorphisms in genes encoding hormones, enzymes, and receptors in these pathways contribute to clinical variability and ethnic differences, offering potential for early diagnosis and personalized medicine. This review summarizes key candidate genes related to insulin metabolism (INS, INSR, IRS-1), the hypothalamic–pituitary–ovarian axis (LHβ, LHCGR, FSHR, GnRHR, AMH, AMHR2, KISS1, CAPN10), steroidogenesis (CYP11A, CYP17A1, CYP19A1, CYP21, 17β-HSD, SHBG, AR, STAR), and other clinically relevant mechanisms such as obesity, lipid metabolism (PPARG, VDR, FTO), and follicular development (ACE).

## 1. Introduction

Polycystic Ovary Syndrome (PCOS) is a common condition in women of reproductive age characterized by menstrual cycle disorders, hyperandrogenism, and polycystic ovarian morphology detected through ultrasound studies [1]. The current diagnosis is established using the modified 2012 Rotterdam criteria, which require the presence of two out of three criteria necessary for diagnosis. This classification determines the emergence of PCOS phenotypes based on the presenting symptomatology, categorized into phenotypes A, B, C, and D [2].

The pathophysiology of this condition is not fully defined; however, several mechanisms have been identified, including dysfunction of the hypothalamic–pituitary–ovarian axis, alterations in steroid synthesis, ovarian cell dysfunction, and abnormalities in insulin metabolism [3].

The prevalence of this condition is high, with estimates suggesting that approximately 6–13% of the population is affected according to World Health Organization data, and about 70% of cases remain undiagnosed clinically [4].

PCOS is a multifactorial condition involving environmental factors such as diet and physical activity, as well as genetic factors and gene–environment interactions, among others [5]. In this context, single-nucleotide polymorphisms (SNPs) have become a target of interest in research that could potentially improve both the diagnosis and personalized treatment of this disease. Genes associated with PCOS are those involved in the pathophysiology of the disorder, particularly those participating in steroid synthesis, insulin metabolism, the hypothalamic–pituitary–ovarian axis, and ovarian function [6]. Studies regarding these genetic variants have yielded controversial results due to several factors, including environmental interactions, the polygenic nature of the disease, significant differences in sample size, and ethnic background [7], which may determine whether the same polymorphism confers risk or protection in different populations.

In this context, this narrative review aimed to synthesize and analyze the available evidence on genetic variants implicated in the pathophysiology of PCOS, as well as potential genetic markers associated with this condition. To achieve this, a search was conducted in the PubMed, Google Scholar, and Scopus databases using keywords such as “Polycystic Ovary Syndrome,” “genetic polymorphism,” “candidate genes,” and “PCOS.” Original research articles, case–control studies, meta-analyses, and reviews evaluating the association between genetic polymorphisms and PCOS were included, with priority given to genes related to steroid synthesis, the hypothalamic–pituitary–ovarian axis, and insulin metabolism.

## 2. Clinical Characteristics and Pathophysiology of PCOS

### 2.1. Diagnostic Criteria for PCOS

Polycystic Ovary Syndrome (PCOS) is a heterogeneous endocrine-metabolic disorder affecting women of reproductive age and is characterized by menstrual irregularity, hyperandrogenism, and polycystic ovarian morphology [1,2,8]. Because of its clinical heterogeneity, diagnostic criteria have evolved over time. Currently, diagnosis is mainly based on the modified Rotterdam criteria, which consider hyperandrogenism, ovulatory dysfunction, and polycystic ovarian morphology, allowing for the identification of four phenotypic presentations of the syndrome [1,2,9,10].

**Figure 1:**
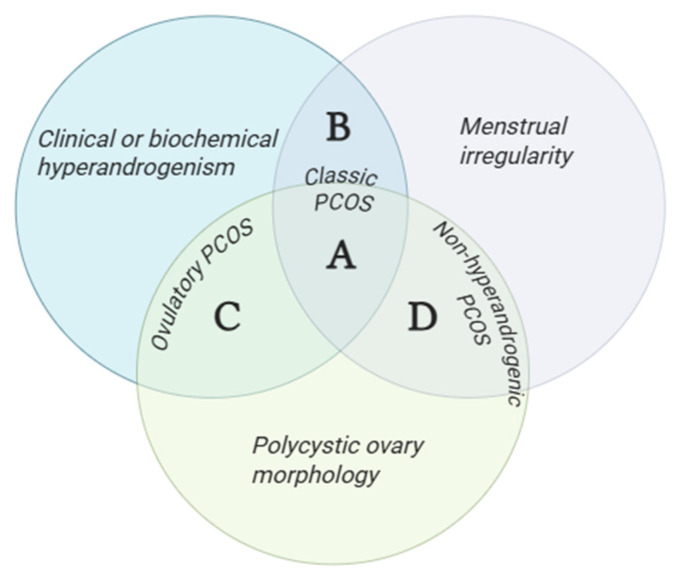
Rotterdam criteria and PCOS phenotypes. Venn diagram illustrating the three Rotterdam diagnostic criteria for polycystic ovary syndrome (PCOS): clinical or biochemical hyperandrogenism, menstrual irregularity (ovulatory dysfunction), and polycystic ovary morphology. The presence of ≥2 of 3 criteria defines four phenotypes: A (classic PCOS), all three features present; B (classic PCOS), hyperandrogenism + menstrual irregularity; C (ovulatory PCOS), hyperandrogenism + polycystic ovary morphology; and D (non-hyperandrogenic PCOS), menstrual irregularity + polycystic ovary morphology.

The diagnostic approach currently used is based on the 2012 modified Rotterdam criteria, and the four phenotypes are defined as follows: A, presence of clinical or biochemical hyperandrogenism, menstrual irregularity, and polycystic ovarian morphology; B, hyperandrogenism and menstrual irregularity; C, hyperandrogenism and polycystic ovarian morphology; and D, the non-hyperandrogenic phenotype, characterized by menstrual irregularity and polycystic ovarian morphology only [11] (Figure 1).

### 2.2. Prevalence and Complications of PCOS

The prevalence of PCOS varies according to the population studied and the diagnostic criteria applied [12]. It is considered one of the most common endocrine disorders in women of reproductive age, with global estimates generally ranging from 6% to 13% [4], although prevalence may be higher when broader criteria such as Rotterdam are used [13]. Available epidemiological data also remain heterogeneous across regions [14,15], particularly in Latin American populations, where studies are still limited [16].

PCOS is associated with important reproductive, metabolic, and psychological complications [6]. The most frequently reported comorbidities include infertility, obesity, insulin resistance, type 2 diabetes mellitus, dyslipidemia, metabolic syndrome, and adverse pregnancy outcomes [1]. In addition, women with PCOS have a higher risk of anxiety, depression, and disordered eating, further supporting the clinical relevance and long-term health burden of the syndrome [9,17].

### 2.3. Pathophysiology

The etiologic mechanisms involved in PCOS have not yet been fully defined, as it is a multifactorial condition driven by the interaction of environmental, metabolic, and hormonal components [9]. Nevertheless, several alterations have been identified, including ovarian dysfunction—particularly in androgen-producing theca cells—as well as disturbances in insulin metabolism, dysregulated steroidogenesis, inflammatory factors, and adipose tissue activity [3].

**Figure 2:**
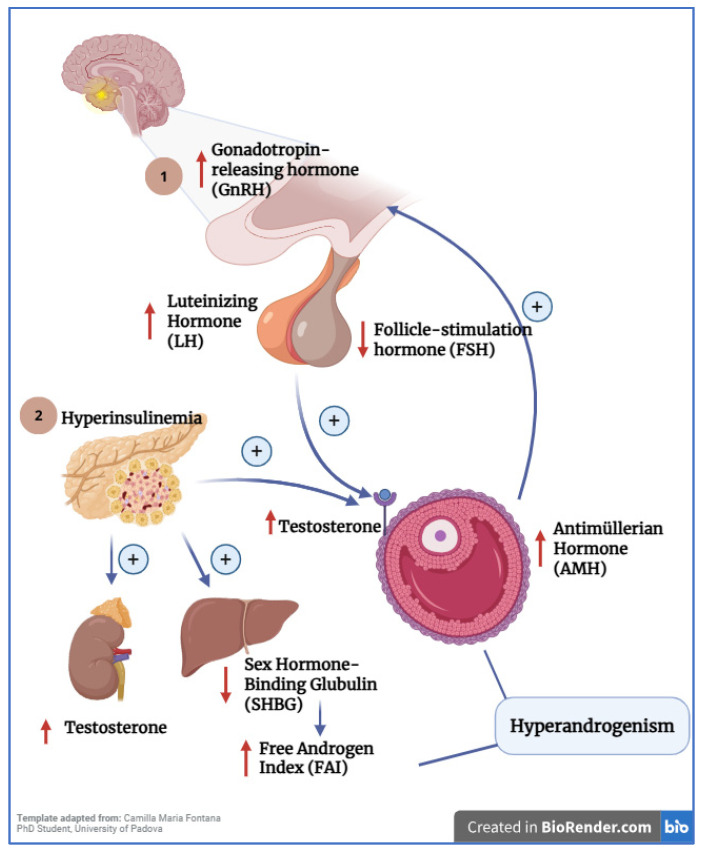
Pathways of the hypothalamic–pituitary–ovarian axis and insulin metabolism involved in PCOS. (1) Alterations in GnRH pulsatility stimulate LH release from the anterior pituitary. Consequently, LH promotes testosterone synthesis within the ovarian theca cells. The accumulation of immature follicles stimulates AMH synthesis. (2) Hyperinsulinemia further triggers androgen production in the adrenal glands and decreases hepatic SHBG synthesis. Also, hyperinsulinemia acts synergistically with LH to stimulate adrenal androgen synthesis and decreases hepatic SHBG synthesis [20]. 1-GnRH, Gonadotropin-Releasing Hormone; 2-LH, Luteinizing Hormone; 3-FSH, Follicle-Stimulating Hormone; 4-AMH, Anti-Müllerian Hormone; 5-SHBG, Sex Hormone-Binding Globulin; 6-FAI, Free Androgen Index. The red arrows indicate changes in hormone levels, while the blue arrows represent the physio-logical pathways that may be involved.

Insulin resistance is one of the most consistently associated mechanisms in PCOS, reported in 65–95% of patients, especially among women with overweight and obesity, although it has also been described in women with normal weight. Insulin suppresses hepatic glucose output while promoting glucose uptake in insulin-sensitive tissues such as skeletal muscle, adipose tissue, the heart, and the liver [18]. In women with PCOS, these tissues exhibit reduced responsiveness despite normal or elevated circulating insulin levels [19]. Hyperinsulinemia synergizes with LH to stimulate theca cells in the ovary, thereby increasing androgen synthesis. Elevated insulin also exerts two additional effects: (1) at the hepatic level, it reduces sex hormone-binding globulin (SHBG), increasing the free androgen index; and (2) at the adrenal level, it stimulates androgen production [18] (Figure 2).

Another key mechanism is dysfunction of the hypothalamic–pituitary–ovarian (HPO) axis, in which increased pulsatile secretion of gonadotropin-releasing hormone (GnRH) has been described in PCOS, leading to elevated secretion of luteinizing hormone (LH) [21]. LH acts directly on ovarian theca cells to enhance androgen synthesis [6]. The accumulation of antral follicles because of hyperandrogenism results in increased anti-Müllerian hormone (AMH) production by granulosa cells, which, in turn, may further stimulate GnRH and LH secretion [20].

Chronic low-grade inflammation has also been implicated in PCOS pathogenesis. Compared with controls, women with PCOS show higher levels of C-reactive protein, interleukin-18 (IL-18), interleukin-6 (IL-6), tumor necrosis factor (TNF-*α*), white blood cell (WBC) count, monocyte chemoattractant protein-1 (MCP-1), and macrophage inflammatory protein-1*α* (MIP-1*α*) [22]. In addition, elevated levels of advanced glycation end products (AGEs) and the receptor for advanced glycation end products (RAGE) have been reported [9].

## 3. Genetics of PCOS

**Figure 3:**
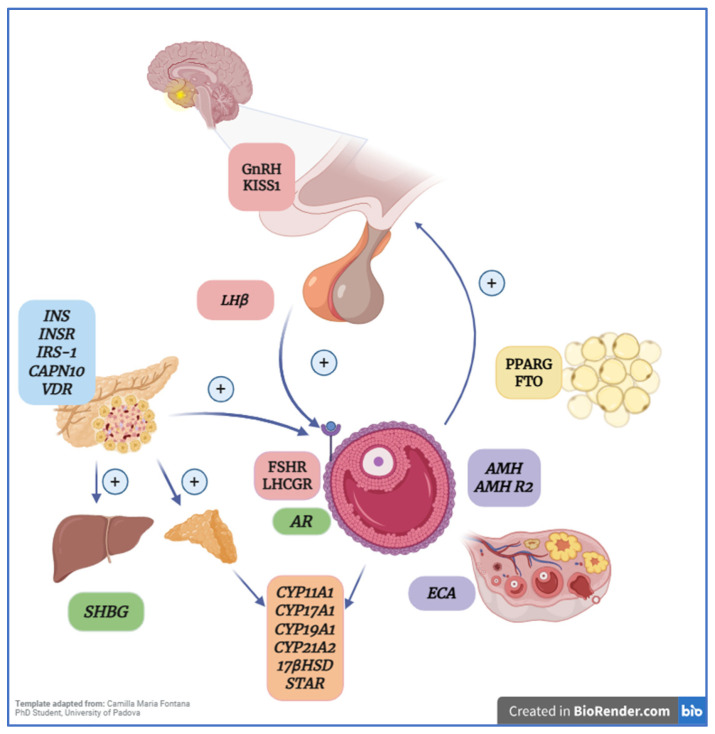
Candidate genes related to etiologic pathways such as steroidogenesis, hypothalamic–pituitary–ovarian (HPO) axis regulation, and ovarian theca cell function. Hypothalamic–pituitary–ovarian axis genes are shown in pink, insulin metabolism in blue, steroidogenesis in orange, follicular development in violet, and obesity/lipid metabolism in yellow. 1-GnRH, Gonadotropin-Releasing Hormone; 2-KiSS-1, Kisspeptin; 3-LH*β*, Luteinizing Hormone Beta Subunit; 4-INS, Insulin; 5-INSR, Insulin Receptor; 6-IRS-1, Insulin Receptor Substrate 1; 7-CAPN10, Calpain 10; 8-VDR, Vitamin D Receptor; 9-FSHR, Follicle-Stimulating Hormone Receptor; 10-LHCGR, Luteinizing Hormone/Choriogonadotropin Receptor; 11-AR, Androgen Receptor; 12-CYP11A1, Cytochrome P450 Family 11 Subfamily A Member 1; 13-CYP17A1, Cytochrome P450 Family 17 Subfamily A Member 1; 14-CYP19A1, Cytochrome P450 Family 19 Subfamily A Member 1; 15-CYP21A2, Cytochrome P450 Family 21 Subfamily A Member 2; 16–17*β*-HSD, 17*β*-Hydroxysteroid Dehydrogenase; 18-STAR, Steroidogenic Acute Regulatory Protein; 19-SHBG, Sex Hormone-Binding Globulin; 20-PPARG, Peroxisome Proliferator-Activated Receptor Gamma; 21-FTO, Fat Mass and Obesity-Associated Gene; 22-AMH, Anti-Müllerian Hormone; 23-AMHR2, Anti-Müllerian Hormone Receptor Type 2; 24-ECA, Angiotensin-Converting Enzyme.

The first studies examining the role of genetics in PCOS were conducted in twins, since heritability can be assessed in the context of relatively similar environmental exposure [23]. In 1997, an Australian study evaluated 19 monozygotic and 15 dizygotic twin pairs and found higher concordance for PCOS among monozygotic twins, supporting a genetic component [24]. Another genetic factor associated with PCOS is the presence of polymorphisms (single-nucleotide variations) in candidate genes related to etiologic pathways such as steroidogenesis, hypothalamic–pituitary–ovarian (HPO) axis regulation, and the function of ovarian theca cells [6] (Figure 3). Regarding genotype–phenotype findings, polycystic ovary syndrome is considered a polygenic and multifactorial disorder; therefore, specific genotypes associated with distinct phenotypes have not been clearly identified. Rather, the interaction between multiple genetic variants and environmental factors determines the level of risk or protection for the development of PCOS (Table 1) [25]. However, specific variants have been associated with particular clinical manifestations of the syndrome, such as hyperandrogenism, which has been linked to variants in the FSHB gene, and insulin resistance, which has been associated with variants in the INSR gene [26]. Current evidence regarding the genetics of PCOS suggests that disease risk depends not only on the presence of risk variants but also on how these variants influence gene expression. In this context, the overexpression of genes such as CYP11A1, CYP17A1, and STAR [27] stimulates ovarian steroid synthesis, whereas reduced expression of SHBG [28] exacerbates androgen availability. Nevertheless, although the evidence is clear and consistent for some genes, it remains limited for others.

Accordingly, genome-wide association studies (GWAS) have substantially reshaped the genetic framework of PCOS by moving the field beyond the methodological and biological limitations inherent to candidate-gene approaches. Large-scale meta-analyses demonstrated that PCOS shares a broadly similar genetic architecture across diagnostic criteria, while also revealing overlap with metabolic traits such as obesity, fasting insulin, type 2 diabetes, lipid levels, and coronary artery disease. More recent multi-ancestry analyses have further expanded the number of susceptibility loci, reinforcing the concept that PCOS is a highly polygenic disorder and that its risk alleles act across both reproductive and metabolic biological domains. Rather than implicating a single pathogenic axis, these studies support the view that PCOS arises from the convergence of genetic determinants involved in gonadotropin secretion and action, folliculogenesis, ovarian steroidogenesis, carbohydrate metabolism, and broader cardiometabolic regulation [23,26,88,89,90].

This broader genome-wide perspective also offers a more informative framework for interpreting the marked heterogeneity of PCOS. Post-GWAS analyses have shown that susceptibility loci can be grouped into partially distinct physiological clusters, including obesity/insulin resistance, hormonal–menstrual regulation, inflammatory or hematologic traits, and other metabolic pathways, supporting the notion that reproductive and metabolic features of PCOS may be driven by overlapping but not identical genetic architectures [91]. In parallel, polygenic approaches, including partitioned polygenic scores, have emerged as promising tools for research-based stratification, helping to link sets of GWAS loci with specific clinical outcomes and comorbidities, although their clinical utility remains preliminary [23,91]. Importantly, follow-up and replication studies have begun to bridge genome-wide discovery with phenotype-level interpretation, showing that GWAS-derived loci such as THADA, INSR, TOX3, DENND1A, PLGRKT, and ZBTB16 may influence insulin resistance, metabolic syndrome, fasting glucose, lipid metabolism, and population-specific susceptibility patterns [92,93,94]. Additional integrative work has also suggested that some established PCOS susceptibility loci may have been shaped by positive selection, which may help explain the persistence and prevalence of PCOS-associated alleles despite their adverse reproductive consequences [95]. Collectively, these findings indicate that candidate-gene evidence remains informative but gains substantially greater biological and translational value when interpreted within the broader context of GWAS, post-GWAS analyses, and emerging polygenic models of PCOS susceptibility [94,96].

### 3.1. Genes Related to Steroid Hormone Synthesis

One etiologic mechanism associated with PCOS is dysregulation of steroidogenesis—a sequential physiological pathway that converts cholesterol into bioactive compounds in steroidogenic organs (the ovary and adrenal cortex) under the influence of multiple enzymes—resulting in increased androgen production [97]. These alterations may be driven by intrinsic factors such as genetic variation or by external influences such as hyperinsulinemia, which is common in these patients [98]. The main enzymes involved in steroid hormone synthesis include hydroxysteroid dehydrogenases (HSDs) and cytochrome P450 (CYP) enzymes [99]. Accordingly, variation in genes affecting the expression of these enzymes may enhance androgen synthesis and, therefore, affect follicular growth and maturation, ultimately contributing to PCOS development [6,100].

#### 3.1.1. CYP11A

CYP11A encodes a cytochrome P450 subfamily member located on chromosome 15q24.1 and mediates the conversion of cholesterol to pregnenolone [100], the first step in steroid synthesis. It is expressed in organs such as the ovaries, testes, kidneys, breasts, and bladder [97]. The first study by Gharani and colleagues in a European population found a significant association between a 5′UTR pentanucleotide repeat polymorphism and hirsutism, as well as with serum testosterone levels in affected and unaffected individuals [29]. A subsequent microsatellite study in Greek women reported a significant association between the CYP11A (tttta)n polymorphism and PCOS, both for PCOS occurrence and for higher total serum testosterone levels [30]. Similar results were reported in a 2014 study from southern India, where CYP11A (tttta)n showed a positive association with PCOS; however, no significant association with clinical manifestations of hyperandrogenism was observed [31]. This microsatellite polymorphism is located in a regulatory region of the gene (−528 bp), and its association with elevated testosterone levels suggests a potential positive regulatory role in the transcription of the CYP11A1 gene. However, expression studies evaluating this VNTR (Variable Number of Tandem Repeats) are still limited, and its definitive regulatory role has not yet been fully established [30,97]. In 2016, a study in an Egyptian cohort (53 PCOS cases and 53 controls) evaluated the rs4077582 polymorphism and reported relevant results for rs4077582 C > T in PCOS using logistic regression adjusted for covariates [32]. A more recent study in Iraqi women found a positive relationship between 5′-UTR (AAAAT) repeats and PCOS, with a higher prevalence of >5 repeats among Iraqi women with PCOS [33].

#### 3.1.2. CYP17A

CYP17A is located on chromosome 10q24.3 and encodes the 17*α*-hydroxylase/17,20-lyase enzyme, which converts pregnenolone to 17-hydroxypregnenolone and, through 17,20-lyase activity, to dehydroepiandrosterone (DHEA) and androstenedione. This gene is expressed primarily in the ovaries and testes, and also in the adrenal cortex [97]. A 2007 study in a Chilean population identified a strong association between the rs743572 T > C polymorphism and PCOS, particularly with metabolic traits such as insulin resistance and BMI [101]. In an Indian study of 200 women, promoter-region polymorphisms in CYP17A were assessed (152 bp PCR product), revealing a significant association of the T > C polymorphism with PCOS and with higher testosterone levels [34]. In a case–control study from Iraq (61 women with PCOS and 30 healthy women), a CYP17A mutation was characterized by two main genotypes: TT (wild type) and TC (heterozygous mutant). Associations were evaluated considering age and BMI, as well as hormonal parameters (testosterone, prolactin, FSH, LH) and metabolic parameters (HDL, LDL, VLDL, triglycerides, cholesterol). Significant findings included lower FSH and HDL in PCOS; however, the polymorphism itself was not significantly associated in this sample [37]. A 2018 study in southern India (250 PCOS cases and 250 controls) assessed rs2414096 and rs700519 in CYP19A1, and rs743572 in CYP17A1, also evaluating lipid profile and BMI, reporting a positive association between the CYP17A1 −34T > C variant and PCOS [35]. In Greece, a study comparing 50 PCOS cases and 50 controls reported a positive association between the homozygous polymorphic allele at −34 T/C and PCOS; additionally, higher testosterone levels were observed in women with the homozygous polymorphic allele compared with heterozygous carriers or non-carriers [102]. A 2020 study in a Pakistani population (204 PCOS cases and 100 controls) found a positive association between the CYP17 5′-UTR MspA1 (TT, TC, CC) polymorphism—particularly the TC genotype—and PCOS; however, no association was found with clinical traits such as infertility [36].

#### 3.1.3. CYP19A1

CYP19A1 encodes aromatase and is located on chromosome 15q21.2. It is expressed in the gonads, adipose tissue, brain, placenta, and bone. In women, aromatase activity is highest in the ovaries, where it catalyzes the conversion of C19 androgens (androstenedione and testosterone) to C18 estrogens (estrone and estradiol) [97]. Alterations in this gene have been associated with aromatase deficiency, manifested by lower estrogen levels and higher testosterone. In an Iraqi case–control study conducted in 2012–2013, including 75 controls and 84 women with PCOS and infertility, the CYP19 rs2414096 polymorphism was analyzed using restriction fragment polymorphism methods, showing a positive association with PCOS susceptibility and with testosterone, LH, and FSH levels [37]. However, a meta-analysis including five case–control studies (1260 PCOS cases and 1030 controls) in Asian populations did not find a significant association between CYP19A1 rs2414096 and PCOS susceptibility under a dominant model [38]. The observed differences in results may be attributed to the sample size, which was considerably smaller in the Iraqi population compared to that of a meta-analysis, which possesses greater statistical power. Another important factor is the diagnostic criteria: the case–control study applied the 2006 Rotterdam criteria, whereas the meta-analysis incorporated studies utilizing all three diagnostic criteria. Furthermore, the meta-analysis focused on women from Asian populations, thereby enhancing both variability and statistical power [37,38].

#### 3.1.4. CYP21

CYP21 encodes 21-hydroxylase and is located on chromosome 6p21.3. The 21-hydroxylase enzyme catalyzes hydroxylation of C21 steroids, converting progesterone and 17-hydroxyprogesterone into 11-deoxycorticosterone and 11-deoxycortisol. This gene is expressed mainly in the adrenal cortex, since the adrenal gland is a key organ for synthesis of steroids such as corticosterone, cortisol, and aldosterone [100]. In a study of Spanish women including 16 with idiopathic hirsutism, 15 with ovarian hyperandrogenism, and 9 with adrenal hyperandrogenism, 8 patients (20%) and one control were heterozygous carriers of CYP21 mutations; the V281L variant was also associated with higher testosterone levels [39]. Conversely, an association study in Italian women with PCOS did not find a significant association between the V281 variant and PCOS risk; however, sample size was small, and larger studies are needed [40].

#### 3.1.5. 17βHSD

The 17*β*HSD gene is located on chromosome 10p14–p15 and is expressed in the ovary and adrenal gland. It encodes 17*β*-hydroxysteroid dehydrogenase, which plays a key role in steroid synthesis by catalyzing the final step in the production of active gonadal steroids—i.e., conversion of androstenedione to testosterone and estradiol [100]. A study in Caucasian women (150 Greek women with PCOS and 51 controls) evaluating a promoter polymorphism at position −71 of the type 5 17*β*-hydroxysteroid dehydrogenase gene did not find a significant association with PCOS; however, it was significantly associated with serum testosterone levels and with a reduced androstenedione/testosterone ratio [41]. In a cohort of White U.S. women with PCOS, five SNPs (rs3763676, rs12529, rs17396032, rs2518049, rs1937841, rs11252946) in 17*β*HSD type 5 were not associated with PCOS risk or testosterone levels [42]. In this case, the observed differences were attributed to the size and heterogeneous composition of the sample. Fifty-nine cases and sixty-seven controls were of Caucasian origin, while the remainder of the sample included individuals of African American, Hispanic, and Asian ancestry [42]. Similarly, in a Chinese case–control study, rs3763676 was not associated with PCOS risk [43].

#### 3.1.6. STAR

STAR encodes the steroidogenic acute regulatory protein, located on chromosome 8p11.2. This protein is essential for transporting cholesterol from the outer to the inner mitochondrial membrane, the first step of steroid biosynthesis. Increased STAR expression has been observed in theca cells from follicles of women with PCOS, suggesting overstimulation of androgen synthesis [100]. A gene-expression study (LHR, STAR, CYP11A, CYP17) in granulosa and theca cells from women with PCOS found overexpression of mRNA for steroidogenic enzymes (CYP11A, CYP17) and for STAR in both granulosa and theca cells compared with controls [27]. A possible mechanism underlying this overexpression is the AKT/LONP1/STAR axis described in the literature. In patients with polycystic ovary syndrome (PCOS), inactivation of the AKT signaling pathway has been reported as a consequence of metabolic disturbances. This inactivation leads to suppression of LONP1, a mitochondrial protease responsible for mitochondrial protein quality control, which, in turn, triggers activation of STAR, ultimately promoting increased steroidogenic activity [103]. In an Iranian case–control cohort evaluating seven STAR polymorphisms (rs104894086, rs104894089, rs104894090, rs137852689, rs10489487, rs104894085), the heterozygous genotype for rs137852689 (amino acid 218 C > T) was present in only seven PCOS patients; thus, results were considered non-significant, and larger studies were recommended [47].

### 3.2. Genes Associated with Insulin Metabolism

Insulin resistance plays a central role in PCOS etiology, with reported prevalence of ~30% in lean women and ~75% in women with obesity who have PCOS [104]. Key genes in insulin signaling include those encoding the insulin receptor (INSR), insulin (INS), insulin receptor substrates (IRS-1, IRS-2), and BMI-related genes such as FTO, TNF, and CAPN [105]. Hyperinsulinemia is a downstream consequence of metabolic alterations associated with PCOS; therefore, genes involved in insulin metabolism may be important in disease progression [6]. In this context, vitamin D is also noteworthy, since women with PCOS commonly present hypovitaminosis D, and vitamin D may activate insulin gene transcription and improve metabolic markers in these patients [106].

#### 3.2.1. INS

Hyperinsulinemia stimulates ovarian theca cells, increasing androgen production via the phosphoinositide 3-kinase/protein kinase B pathway. INS is a “sandwich” gene located at 11p15.5 between tyrosine hydroxylase and IGF-II [6]. The INS-VNTR tandem repeat polymorphism has been associated with diabetes mellitus risk, cardiovascular disease, and PCOS risk. A study in Korean women with PCOS found no significant association between INS-VNTR and PCOS in that population [48]. A hospital-based case–control study in India (169 PCOS cases and 169 controls) also reported no significant association between INS polymorphisms and PCOS [49]. A meta-analysis including 13 case–control studies (1767 cases and 4108 controls) found no significant association between INS-VNTR and PCOS in the general population; however, this variant might be associated with anovulatory PCOS, pending confirmation in larger studies [50].

#### 3.2.2. INSR

INSR encodes the insulin receptor and is located on chromosome 19. In a study of lean Caucasian women (99 women and 136 controls), a significant association was reported between a C/T SNP at His1058 in INSR and PCOS among lean women compared with lean healthy controls [51]. These findings were consistent with results from a Chinese population study that assessed body composition and hormonal parameters and found a positive association, particularly in non-obese women with PCOS [52]. In contrast, a Korean study (174 PCOS cases and 93 controls) did not find a significant association between this polymorphism and PCOS [53]. Additional variants linked to PCOS risk include a T/C change at Cys1008 in exon 17, identified in a Chinese population, where the homozygous mutant CC allele was more frequent among PCOS patients [54]. A 2015 meta-analysis found a significant association for rs2059807, but no association for rs1799817/rs2059806 in PCOS [55]. Some limitations observed in this study included reduced statistical power in the BMI subgroup analysis, the lack of calculation of the odds ratio for rs2059807, the inability to determine the ethnicity of participants in some countries, and variability in the cutoff values used to define leanness and obesity among the analyzed studies. Additionally, the control groups were heterogeneous (including infertile women, older women, and healthy women), and different diagnostic criteria were used across studies [55].

#### 3.2.3. IRS-1

Insulin signaling begins when insulin binds its receptor, followed by autophosphorylation of the *β* subunit and phosphorylation of insulin receptor substrates (IRS-1, IRS-2) through tyrosine kinase activity. Given the close relationship between insulin metabolism disturbances and PCOS pathophysiology, genetic variation in IRS-1 may influence PCOS risk [6]. In a Chilean study (82 women with PCOS and 70 controls), a higher frequency of the G972R polymorphism was observed in PCOS, along with higher 2 h post-stimulation insulin levels [56]. Similar findings were reported by Dilek and colleagues in Turkish women, where G972R frequencies were higher in PCOS; carriers also showed higher rates of obesity, insulin resistance, and elevated fasting insulin, while free androgen levels were not associated with genotype [57]. In a cohort from southern India analyzing INS, INSR, IRS1, IRS2, PPAR-G, and CAPN10 variants, a significant association was found for IRS-1 Gly972Arg, whereas no significant associations were observed for the other genes [49].

#### 3.2.4. Calpain 10 (CAPN10)

CAPN10 is located on chromosome 2q37.3 and encodes a calcium-dependent cysteine protease involved in insulin action. Variants in this gene have been linked to insulin metabolism disturbances and increased risk of type 2 diabetes mellitus [6]. A Spanish case–control study (55 PCOS cases and 93 controls) found a significant association with the UCSNP-44 polymorphism [58]. Another Spanish study evaluating CAPN10 haplotypes and PCOS phenotypes analyzed 482 CAPN10 haplotypes and confirmed the UCSNP-44 association [59]. In Caucasian women genotyped for three CAPN10 polymorphisms, comparing idiopathic hirsutism vs. hirsutism due to hyperandrogenism or PCOS, a significant association was found between the uncommon C allele in UCSNP45 and idiopathic hirsutism, while the A allele in UCSNP43 was associated with higher hirsutism scores (Ferriman–Gallwey) [60]. A meta-analysis including 11 studies found that the homozygous and recessive models for UCSNP-63 were significantly associated as protective factors for PCOS; additionally, the insertion allele and recessive model for UCSNP-19 were also identified as protective [61]. These SNPs, although located in intronic regions and not affecting the amino acid structure of the protein, may still have an effect on gene expression or on alternative splicing mechanisms. CAPN10 facilitates the translocation of GLUT-4 transporters and glucose uptake in skeletal muscle and adipocytes, influencing insulin sensitivity [107].

#### 3.2.5. VDR

VDR encodes the vitamin D receptor and is located on chromosome 12q13.11. VDR is a nuclear receptor expressed in multiple tissues, including the intestine, kidney, parathyroid, pancreatic beta cells, bone, ovarian tissue, and endometrium [100]. A study in southern India (95 PCOS patients and 130 controls) assessed three VDR polymorphisms—BsmI A/G (rs1544410), ApaI A/C (rs7975232), and TaqI T/C (rs731236)—using PCR-RFLP, reporting higher prevalence of these genotypes and of the BsmI G, ApaI C, and TaqI C alleles in PCOS patients compared with controls [77]. An Iranian study (35 PCOS cases and 35 controls) reported a significant association of BsmI and ApaI with PCOS risk, and heterozygous genotypes appeared to reduce susceptibility [78]. Conversely, in Turkic-Azeri Iranian women with PCOS, no significant association was found for VDR exon 2 FokI (rs10735810) or intron 8 BsmI (rs1544410) [79]. These results aligned with an Austrian study that also assessed metabolic/endocrine markers including 25(OH)D levels and found no association between VDR variants (Cdx2, Bsm-I, Fok-I, Apa-I, Taq-I) and PCOS risk [80]. Some confounding factors involved may have an effect on the results obtained in these studies. Among them, we can mention several modulators of VDR activity, such as circulating vitamin D levels, dietary habits, sun exposure, and body mass index (BMI). Vitamin D exerts its critical functions through its active form, 1,25-dihydroxyvitamin D3 (1,25(OH)_2_D_3_), by binding VDR and regulating endocrine and metabolic functions [108].

On the other hand, inconsistent results may be due to false positives, environmental factors, variation in the statistical methods used, or differences in population allele frequencies [78].

### 3.3. Genes Involved in the Hypothalamic–Pituitary–Ovarian Axis

Dysregulated gonadotropin-releasing hormone (GnRH) secretion at the hypothalamic level has been reported in PCOS, directly affecting gonadotropin action; gonadotropins are glycoprotein hormones secreted by the anterior pituitary [5]. These include luteinizing hormone (LH), follicle-stimulating hormone (FSH), and human chorionic gonadotropin (hCG), all of which play critical roles in the menstrual cycle, follicular development, and maturation; dysregulation contributes to anovulation and polycystic morphology [6]. Key genes associated with this mechanism are summarized below.

#### 3.3.1. LH

LH is central to PCOS pathophysiology: elevated LH in affected patients stimulates androgen synthesis and impairs follicular maturation, contributing to anovulatory dysfunction. Genetically, LH alterations may be linked to variants in the gene encoding the *β* subunit of this hormone [6]. In a Japanese cohort, two nonsense variants (T(986)-C and T(1008)-C) and five silent mutations (C(894)-T, G(1018)-C, C(1036)-A, C(1098)-T, C(1423)-T) were analyzed in the *β*-subunit gene. The highest frequency of the novel allele was observed for C(1036)-A and was significant among women with PCOS, endometriosis, premature ovarian failure, and luteal insufficiency [62]. In northern India, the rs1056917 polymorphism (serine-to-glycine substitution) in the *β* subunit was associated with PCOS and with clinical/biochemical parameters; women with the heterozygous TC genotype had higher PCOS risk than those with TT [63].

#### 3.3.2. LHCGR

LHCGR encodes the luteinizing hormone/choriogonadotropin receptor and is located on chromosome 2p16.3. It is expressed mainly in granulosa cells, mediating ovulation in response to LH surges [100]. In a European cohort study (905 PCOS cases and 956 controls), rs7562215 was associated with PCOS after adjustment for ethnicity, age, and BMI; several markers near rs10495960 also showed association [64]. In India, rs4953616 and rs7371084 were analyzed, with rs4953616 significantly more frequent in PCOS; the TT mutant genotype conferred 1.77-fold higher risk [65]. However, a meta-analysis assessing rs2293275 reported no statistically significant differences in PCOS risk across Arab, Asian, and Caucasian populations, highlighting etiologic complexity [66].

#### 3.3.3. FSHR

FSHR encodes the follicle-stimulating hormone receptor and is located on chromosome 2p21; it is expressed in granulosa cells similarly to LHCGR. FSHR and its interaction with FSH are directly related to oogenesis, gametogenesis, and follicular maturation [100]. A genome-wide association study in European populations identified a strong association between rs1922476 and PCOS, as well as the coding SNP rs6165 (Ala → Thr) in FSHR [64]. In a Chinese Han population study (744 PCOS cases and 895 controls), significant associations were found at three loci: rs13405728, rs13429458, and rs2479106. Another Shanghai-based Chinese study, including PCOS (60), controls (92), and premature ovarian insufficiency (40), reported positive associations between Thr307Ala (rs6165), Asn680Ser (rs6166), and PCOS [67].

#### 3.3.4. GnRHR

GnRHR encodes the GnRH receptor, which influences LH and FSH synthesis and secretion. It is located in the anterior pituitary and in extra-pituitary tissues such as the placenta, ovaries, breasts, and cancerous tissue [100]. A study in PCOS patients undergoing infertility treatment with IVF-ET reported a higher frequency of CC + CT haplotypes (rs12644822, rs3756159, rs13138607) in PCOS; patients carrying CC + CT at all three positions had lower pregnancy rates [68]. In 2017, a whole-genome study in a consanguineous family with three sisters diagnosed with PCOS identified rs104893836 in the coding exon of GnRHR, homozygous in affected sisters and heterozygous in the parents [69].

#### 3.3.5. KISS

Kisspeptin is located on chromosome 1q32, and KISS1 activates a G protein–coupled transmembrane receptor (GPR54) in GnRH neurons, increasing LH production. Kisspeptin is crucial for HPO axis regulation and participates in sex-steroid feedback on GnRH production, influencing physiological processes such as sexual maturation and reproductive activity during lactation [100,109]. In women with hypothalamic amenorrhea, kisspeptin-54 supplementation significantly increased LH pulsatility [110]. Elevated kisspeptin levels have also been reported in PCOS; for example, Umit and colleagues recruited 157 patients and found serum kisspeptin levels were inversely related to FSH and directly related to LH [111]. Another study evaluating the temporal association between episodic kisspeptin and LH secretion found coupling only in eumenorrheic patients (intermenstrual interval <45 days), whereas patients with oligomenorrhea did not show this temporal coupling [112]. A recent meta-analysis on KISS1 variants rs4889 and rs372790354 in Asian populations found a significant association between rs372790354 and PCOS risk under both allelic and dominant models [73]. Conversely, a Chinese cohort did not find a significant association between KISS1 variants and PCOS risk but did find associations between CC genotypes of rs4889 and AA genotypes of rs5780218 with higher estradiol levels, and the mutant G allele was found to be strongly associated with the polymorphism in the GPR54 gene (rs10407968) in patients with PCOS [74].

### 3.4. Genes Associated with Steroid Hormone Action

Steroid hormones exert biological effects via receptors that mediate signaling in target tissues. In this context, sex hormone-binding globulin (SHBG) binds testosterone and regulates its availability in tissues and its circulating concentration; androgen receptors mediate androgen action in multiple organs [6].

#### 3.4.1. SHBG

SHBG is located on chromosome 17p13.1 and encodes sex hormone-binding globulin, synthesized in the liver. Its primary function is the high-affinity binding of testosterone and estrogens, regulating their bioavailability in target tissues. Women with PCOS commonly present hyperandrogenism and insulin resistance with hyperinsulinemia; elevated insulin inhibits hepatic SHBG secretion, contributing to increased free androgens [100]. In a case–control study (180 women with PCOS and 168 controls), the SHBG (TAAAA)n polymorphism showed six alleles (6–11 repeats) in both groups, with longer alleles (>8 repeats) more frequent in PCOS [28]. In an Indian cohort evaluating rs6259, no significant differences were found in PCOS risk or SHBG levels [44]. A possible explanation for this result is that the D327N polymorphism does not directly affect the affinity of SHBG for steroids; rather, it creates an additional glycation site at the N-terminal end, which may increase the protein’s half-life and reduce its plasma clearance [44].

#### 3.4.2. AR

AR is located on the X chromosome (Xq11–12) and encodes the androgen receptor [100]. Androgen receptors are closely linked to PCOS, since androgenic effects underlying the clinical features of PCOS are mediated through receptor activity. A CAG repeat polymorphism has been identified that modulates androgen receptor activity. In a U.S. study comparing 72 PCOS patients and 179 controls, PCOS patients had a shorter mean CAG repeat length, which is inversely related to receptor activity [45]. Similar findings were observed in a Chinese-ancestry sample (261 PCOS patients and 278 controls), where shorter alleles were more frequent in the PCOS group than in controls [46].

### 3.5. Genes Associated with Follicular Development

This category includes anti-Müllerian hormone (AMH) and angiotensin-converting enzyme (ACE). AMH is elevated in women with PCOS and is an important marker in fertility studies; it is also a potential candidate to substitute follicle count in PCOS diagnosis, particularly when ultrasound criteria are not clearly defined [113]. ACE is a key component of the renin–angiotensin–aldosterone system (RAAS) and is expressed in multiple tissues, including the ovary. RAAS is involved in oocyte maturation, ovulation, corpus luteum formation, and adrenal/ovarian steroid synthesis [114].

#### 3.5.1. AMH

Genetic variants associated with PCOS include AMH rs10407022 (Ile49Ser) and its type II receptor AMHR2 rs2002555. A study in 331 women with PCOS and 3635 population controls reported that Ser carriers had a lower prevalence of polycystic morphology and lower follicle counts due to reduced hormone activity; however, no statistically significant association was found between PCOS risk and these variants [70]. In a recent cohort of women of Northern European ancestry, rs10406324 in the AMH promoter was associated with lower serum AMH levels in carriers, but no association with follicle count or clinical parameters was found [71]. These findings suggest that genetic differences within the promoter region may lead to differential regulation of AMH expression in patients with similar phenotypic characteristics [71]. A sequencing study in European women (608 PCOS patients) identified 20 additional variants in/near AMH and AMHR2, for a total of 37 variants among PCOS patients; a second cohort of non-Finnish European ancestry was also analyzed [72].

#### 3.5.2. ACE

ACE encodes angiotensin-converting enzyme, which converts angiotensin I to angiotensin II and is expressed in multiple organs, including the ovary. ACE is important for the regulation of fluid balance and blood pressure and, in the ovary, contributes to follicular development, oocyte maturation, and ovulation [100]. The PCOS-associated variant is an insertion/deletion in intron 16. A Greek case–control study found a significant increase in the frequency of the polymorphic genotype among PCOS patients compared with controls [84]. In Turkish and U.S. cohorts, no significant association with PCOS risk was found, although the D allele and DD genotype were associated with higher frequency of insulin resistance among women with PCOS [85,86]. The differences observed in this study’s findings were attributed to the small sample size, the lack of comparison between phenotypic and allelic frequencies in lean and obese women, and the absence of an oral glucose tolerance test. Nevertheless, the authors recommended confirming these results through larger prospective and controlled studies [85]. A meta-analysis of 13 studies evaluating the ACE I/D polymorphism and insulin resistance in PCOS found that the D allele promotes PCOS development, and the I/D variant was associated with insulin-resistant PCOS in Asian women [87].

### 3.6. Genes Associated with Obesity and Lipid Metabolism

Obesity and PCOS are closely linked (38–88% of patients), and type 2 diabetes mellitus shares physiopathological mechanisms with PCOS. Several candidate genes have been identified, including fat mass and obesity-associated gene (FTO) due to its relationship with obesity and T2DM [115]. The peroxisome proliferator-activated receptor gamma (PPARG), a transcription factor regulating genes involved in glucose and lipid metabolism, is also relevant [116].

#### 3.6.1. PPARG

PPARG is located on chromosome 3p24.2–p25 and plays a key role in lipid metabolism by participating in adipocyte differentiation [100]. One of the most studied polymorphisms associated with abdominal obesity in Korean women is Pro12Ala [117]. In a study of 100 women with PCOS and 120 age-matched controls, the Ala allele was more frequent among PCOS patients; carriers also had higher body composition and metabolic parameters than non-carriers [75]. In a German study of 102 PCOS patients (NIH criteria) and 104 controls, 22.5% of patients carried at least one Ala allele (X/Ala) and showed greater insulin sensitivity and lower hirsutism scores [76]. However, a Greek study including 180 women with PCOS found no significant association for Pro12Ala distribution between PCOS and controls, and no differences in hormonal or metabolic profile were observed [7]. The differences in the results of the studies may be explained by the genetic background of the populations investigated, as well as by uncontrolled environmental factors, including diet and physical activity. In this regard, fatty acids have been identified as natural ligands of PPAR-γ, although their affinity depends on chain length and degree of saturation [118]. Additionally, Ala12 carriers have been reported to be more sensitive to moderate-to-vigorous aerobic exercise [119].

#### 3.6.2. FTO

FTO is expressed in most tissues, is located on chromosome 16q12.2, and is associated with BMI and obesity [100]. A Brazilian study (199 PCOS patients and 99 women without hirsutism with regular ovulatory cycles) assessed intron 1 polymorphisms rs9939609 and rs8050136 and found non-significant results for these polymorphisms and haplotypes with respect to PCOS [81]. In this regard, the authors point out the sample size (300 participants) as a limitation. However, BMI was not adjusted for, which represents a key confounding factor in studies evaluating the FTO gene [81]. A meta-analysis evaluating FTO rs9939609 and PCOS found no significant association in the general population but suggested a possible direct association in East Asian PCOS patients independent of BMI [82]. Regarding the meta-analysis, some of the included studies did not control for potential confounding factors, such as BMI-adjusted data. Furthermore, the genetic background may vary according to the PCOS phenotype when applying the Rotterdam criteria. In this context, most studies did not specify the number of participants within each phenotype [82]. A UK study in women of British/Irish ancestry found a significant association between FTO rs9939609 genotype and obesity among women with PCOS [83].

## 4. Conclusions

PCOS is an endocrine–metabolic disorder with a high prevalence among women of reproductive age (approximately 5–10%, depending on the diagnostic criteria used) and has been linked to serious health complications, including diabetes mellitus, cardiovascular disease, obesity, metabolic syndrome, and infertility. From a quality-of-life and mental health perspective, it is a condition that can substantially impair psychological well-being, frequently contributing to eating disorders, depression, low self-esteem, and even suicide attempts. Therefore, early diagnosis and preventive strategies are essential to reduce long-term complications, and in this context, genetics, particularly candidate genes, offers a valuable framework for understanding the biological diversity and pathophysiological complexity of the syndrome.

In this narrative review, we compiled the main genes associated with key etiologic pathways involved in PCOS, including insulin metabolism (INS, INSR, IRS-1), the hypothalamic–pituitary–ovarian axis (LHβ, LHCGR, FSHR, GnRHR, AMH, AMHR2, KISS1, CAPN10), and steroidogenesis (CYP11A, CYP17A1, CYP19A1, CYP21, 17βHSD, SHBG, AR, STAR). We also included genes implicated in additional pathways relevant to PCOS, such as obesity and lipid metabolism (PPARG, VDR, FTO) and follicular development (ACE). Collectively, these findings support the concept that PCOS arises from the interaction of multiple reproductive, metabolic, and endocrine pathways, rather than from disruption of a single molecular axis.

Although numerous studies have explored candidate genes and their relationship with PCOS, many findings remain inconclusive, sample sizes vary widely, and most available evidence comes from European, North American, Asian, and Middle Eastern populations. Consequently, further research in Latin populations is needed, as genetic expression is influenced by ethnic, epigenetic, and environmental factors that differ across populations. Importantly, PCOS cannot be explained by the effect of a single gene; rather, it is a multifactorial and polygenic condition. Taken together, the current evidence indicates that the genetic basis of PCOS is complex, heterogeneous, and not yet sufficiently robust to support definitive clinical implementation. This complexity should be considered in future research aimed at improving early diagnosis and advancing personalized medicine strategies.

From a clinical perspective, the available genetic evidence may contribute, in the future, to a better stratification of women at risk of developing PCOS or of presenting specific reproductive and metabolic complications, such as insulin resistance, obesity, dyslipidemia, infertility, or a more severe hyperandrogenic phenotype. Although the routine clinical use of genetic markers, GWAS-derived loci, or polygenic risk scores is still limited, these tools represent promising approaches for refining risk prediction, improving phenotypic classification, and identifying subgroups of patients who may benefit from earlier monitoring or more targeted interventions. However, at present, genetic testing has no established utility for the routine diagnosis or clinical management of PCOS.

In translational terms, future studies should prioritize large and ethnically diverse cohorts, standardized diagnostic criteria, and the integration of candidate-gene findings with GWAS, post-GWAS analyses, and polygenic approaches. Likewise, functional studies are required to clarify the biological relevance of associated variants and their interaction with environmental and epigenetic factors. Advancing toward the combined use of genetic, metabolic, endocrine, and clinical markers may help build more robust predictive models and support the development of precision medicine approaches for PCOS in the coming years. In conclusion, the available evidence supports that PCOS has a complex and polygenic genetic architecture; nevertheless, its true translational and clinical value will depend on more integrative, functionally validated, and clinically reproducible research before genetic information can be meaningfully incorporated into routine practice.

## Figures and Tables

**Table 1:** Polymorphisms of candidate genes associated with the pathophysiology of PCOS.

Gene	Chromosome	Pathway	Encodes/Function	Main Polymorphisms	Relationship to PCOS	Population	References
**CYP11A1**	15q24.1	Steroid synthesis	Conversion of cholesterol to pregnenolone (first step of steroidogenesis)	rs4077582	Positive association with PCOS and ↑ testosterone	European, South Asian, Middle Eastern	[29,30,31,32,33]
**CYP17A1**	10q24.3	Steroid synthesis	17*α*-hydroxylase/17,20-lyase (androgen synthesis)	rs743572	Positive association with PCOS and ↑ testosterone; variable results	Latin American, South Asian, European	[30,34,35,36]
**CYP19A1**	15q21.2	Steroid synthesis	Aromatase (androgens → estrogens)	rs2414096	Positive association in some studies; meta-analysis not significant	Middle Eastern, Asian	[37,38]
**CYP21**	6p21.3	Steroid synthesis	21-hydroxylase (cortisol/aldosterone synthesis)	rs6471	Associated with hyperandrogenism; inconclusive for PCOS	European	[39,40]
**17*β*HSD (type 5)**	10p14–p15	Steroid synthesis	Conversion of androstenedione → testosterone	rs12529 rs3763676 and others	Not associated with PCOS; yes with testosterone levels	European, North American, East Asian	[41,42,43]
**SHBG**	17p13.1	Steroid action	Sex hormone transport	rs35785886 rs6259	Long alleles associated with PCOS; rs6259 not significant	European, South Asian	[28,44]
**AR**	Xq11–12	Steroid action	Androgen receptor	rs3032358	Short repeats associated with PCOS	North American, East Asian	[45,46]
**STAR**	8p11.2	Steroid synthesis	Mitochondrial cholesterol transport	rs137852689 and others	Overexpression in PCOS; SNPs not conclusive	European, Middle Eastern	[27,47]
**INS**	11p15.5	Insulin metabolism	Insulin	rs689	No clear association; possible anovulatory PCOS	East Asian, South Asian, Multiethnic	[48,49,50]
**INSR**	19p13	Insulin metabolism	Insulin receptor	rs1799817 rs2059807	Association in non-obese PCOS; variable results	European, East Asian, Multiethnic	[51,52,53,54,55]
**IRS-1**	2q36	Insulin metabolism	Insulin signaling	rs1801278	Association with PCOS, insulin resistance, and hyperinsulinemia	Latin American, Middle Eastern, South Asian	[49,56,57]
**CAPN10**	2q37.3	Insulin metabolism	Insulin action	rs2975760 rs3842570	Association and protective effects	European, Multiethnic	[58,59,60,61]
**LH*β***	19p13.3	H-H-O axis	LH *β* subunit	rs1056917 and point mutations	Association with PCOS and ovulatory alterations	East Asian, South Asian	[62,63]
**LHCGR**	2p16.3	H-H-O axis	LH/hCG receptor	rs7562215, rs4953616	Positive association in some populations	European, South Asian, Multiethnic	[64,65,66]
**FSHR**	2p21	H-H-O axis	FSH receptor	rs6165, rs6166	Consistent association with PCOS	European, East Asian	[64,67]
**GnRHR**	4q13.2	H-H-O axis	GnRH receptor	rs12644822, rs104893836	Associated with PCOS and lower pregnancy rate	East Asian, European	[68,69]
**AMH/AMHR2**	19p13/12q13	Follicular development	Anti-Müllerian hormone	rs10407022, rs10406324	Modulates AMH levels; no clear direct risk	European, non-European	[70,71,72]
**KISS1**	1q32	H-H-O axis	Regulation of HPO axis	rs4889, rs372790354	Recent association with PCOS	Middle Eastern, East Asian, European	[73,74]
**PPARG**	3p25	Obesity and Lipid Metabolism	Adipocyte differentiation	rs1801282	Variable results; metabolic influence	East Asian, European	[7,75,76]
**VDR**	12q13.11	Insulin metabolism	Vitamin D receptor	rs1544410, rs7975232 rs731236 rs10735810	Population-dependent association	South Asian, Middle Eastern, European	[77,78,79,80]
**FTO**	16q12.2	Obesity and Lipid Metabolism	Regulation of BMI and obesity	rs9939609	Associated with obese PCOS and East Asian populations	Latin American, Asian, European	[81,82,83]
**ECA (ACE)**	17q23	Follicular development	Renin-angiotensin system	rs4646994	Association with PCOS-IR in Asians	European, Middle Eastern, North American, Asian	[84,85,86,87]

1-GnRH, Gonadotropin-Releasing Hormone; 2-KiSS-1, Kisspeptin; 3-LH*β*, Luteinizing Hormone Beta Subunit; 4-INS, Insulin; 5-INSR, Insulin Receptor; 6-IRS-1, Insulin Receptor Substrate 1; 7-CAPN10, Calpain 10; 8-VDR, Vitamin D Receptor; 9-FSHR, Follicle-Stimulating Hormone Receptor; 10-LHCGR, Luteinizing Hormone/Choriogonadotropin Receptor; 11-AR, Androgen Receptor; 12-CYP11A1, Cytochrome P450 Family 11 Subfamily A Member 1; 13-CYP17A1, Cytochrome P450 Family 17 Subfamily A Member 1; 14-CYP19A1, Cytochrome P450 Family 19 Subfamily A Member 1; 15-CYP21A2, Cytochrome P450 Family 21 Subfamily A Member 2; 16–17*β*-HSD, 17*β*-Hydroxysteroid Dehydrogenase; 18-STAR, Steroidogenic Acute Regulatory Protein; 19-SHBG, Sex Hormone-Binding Globulin; 20-PPARG, Peroxisome Proliferator-Activated Receptor Gamma; 21-FTO, Fat Mass and Obesity-Associated Gene; 22-AMH, Anti-Müllerian Hormone; 23-AMHR2, Anti-Müllerian Hormone Receptor Type 2; 24-ECA, Angiotensin-Converting Enzyme, 25-H-H-O axis: Hypothalamic–Pituitary–Ovarian Axis. The upward arrows indicate an increase, and the downward arrows indicate a decrease.

## Data Availability

No new data were created or analyzed in this study.

## Abbreviations

The following abbreviations are used in this manuscript:

CYP11A1	Cytochrome P450 family 11 subfamily A member 1
CYP17A1	Cytochrome P450 family 17 subfamily A member 1
CYP19A1	Cytochrome P450 family 19 subfamily A member 1
CYP21A2	Cytochrome P450 family 21 subfamily A member 2
17*β*HSD	17*β*-hydroxysteroid dehydrogenase
SHBG	Sex hormone binding globulin
AR	Androgen receptor
INSR	Insulin receptor
FSHR	Follicle-stimulating hormone receptor
GnRHR	Gonadotropin-releasing hormone receptor
LHCGR	Luteinizing hormone/choriogonadotropin receptor
AMH	Anti-Müllerian Hormone
AMHR2	Anti-Müllerian Hormone Receptor Type 2;
PPARG	Peroxisome proliferator-activated receptor gamma
VDR	Vitamin D receptor
FTO	Fat mass and obesity associated protein
ACE	Angiotensin converting enzyme
H-H-O	Hypothalamic–Pituitary–Ovarian
AES	Androgen Excess Society
NIH	National Institutes of Health
CAH	Congenital adrenal hyperplasia
RAAS	Renin–angiotensin–aldosterone system
21-OH	21-hydroxylase
ACTH	Adrenocorticotropic hormone
OCP	Oral contraceptive
PCOM	Polycystic ovarian morphology
